# Supplementary material for: MetaRibo-Seq measures translation in microbiomes
Source: Nat Commun. 2020 Jun 29;11:3268. doi: 10.1038/s41467-020-17081-z (PMC7324362; doi:10.1038/s41467-020-17081-z)
Supplement: Supplementary file 10 — Supplementary Data 7 [file 41467_2020_17081_MOESM10_ESM.zip › File2/Confidence_VeryHigh_Taxonomy/346846_out.krona.html]

Javascript must be enabled to view this page.

members
magnitude
magnitudeUnassigned
count
unassigned
taxon
rank

346846\_out

7

2
superkingdom
3

phylum
3
1239

186801
class
3

3
order
186802
1

SRS104636\_contig\_number\_24434

1
family
186806

1730
1
genus

39496
1
species

SRS015217\_contig\_number\_13647

186803
family
1


SRS019161\_contig\_number\_41369
1
species
877421

4

SRS013965\_contig\_number\_contig-100\_934.47919SRS063040\_contig\_number\_contig-100\_8989.49491SRS098571\_contig\_number\_6304SRS104311\_contig\_number\_contig-100\_36205.64675
